# On the Comparative Mortality of Large Towns and Rural Districts, and the Causes by Which It Is Influenced

**Published:** 1855-12

**Authors:** John Snow


### PAPERS AND COMMUNICATIONS.

---

### ON THE COMPARATIVE MORTALITY OF LARGE TOWNS AND RURAL DISTRICTS, AND THE CAUSES BY WHICH IT IS INFLUENCED. By JOHN SNOW, M.D.

[Read before the Epidemiological Society, on May 2, 1853].

It is well known that the average duration of life in the large towns of this country is much less than amongst the rural population. This depends, as most persons are aware, partly on the smaller number of persons who attain to old age in large towns, and partly on the greater mortality in infancy and early childhood. For instance, rather more than forty per cent. of the deaths which take place in London occur under five years of age; nearly forty-eight per cent. of the deaths in Birmingham, fifty-one per cent. in Manchester, and fifty-two per cent. in Liverpool, occur before the same age; consequently more than half the children that are born in the last two towns die before they are five years old. In Wiltshire, on the other hand, the deaths under five years comprise but thirty-one and a-half per cent. of the deaths at all ages; in Surrey, not including the metropolitan part of it, the deaths under five are not quite thirty-one per cent. of the whole; and in the united district of Guildford, Farnham, and Hambledon, in the more distant and rural part of Surrey, the deaths under five years do not quite reach twenty-nine per cent. of the whole number of deaths. When the deaths under five years of age are considered, in relation to the population of the districts in which they occur, the difference between the large towns and rural districts appears still more striking. For instance, the deaths during this period of life in Liverpool are 176 annually for each 10,000 of the inhabitants; whilst in Surrey they are only 58, or less than one-third the number.

It is perhaps not very generally known that, at one period of life, a rule pretty generally obtains just the reverse of that considered above; and that the mortality is greater in the rural districts than amongst the inhabitants of London and certain other large towns.

On glancing the eye over the table below, showing the mortality of London, as contrasted with that of the extra-metropolitan part of Surrey, at various periods of life, it will be observed that the mortality of the country population rises at a certain period above that of the town. In the first part of the table, in which the deaths at different ages are compared with the total mortality, the proportion of deaths in Surrey begins at five years to rise above that of London, and continues above it to the age of twenty-five. Amongst females, this elevation of the mortality in Surrey is greater than amongst males. In the second part of the table, in which the mortality at different ages is compared with the entire population, the deaths from ten to fifteen are higher for both sexes in Surrey than in London, and from fifteen to twenty-five they are higher for the female sex. After the age of twenty-five the mortality of London rises again above that of Surrey, and continues above it till about sixty or seventy years of age, when it falls again, on account of the smaller number of persons who attain to old age in the metropolis. The greatest number of deaths amongst males in any decennial period, after the age of five years, occur in London between thirty-five and forty-five years of age; whilst in Surrey they occur between sixty-five and seventy-five, or thirty years later. Amongst females, however, the greatest mortality in any decennial period, after the age of five, takes place in London from sixty-five to seventy-five, and in Surrey from seventy-five to eighty-five, only ten years later.

### Annual Mortality in Seven Years, 1838–1844.

### No. I.—In 100,000 Deaths at all Ages.

| | London. | | | Surrey. | |
|---|---|---|---|---|---|
| *Ages.* | *Males.* | *Females.* | *Ages.* | *Males.* | *Females.* |
| 0 to 1 | 21,770 | 18,300 | 0 to 1 | 18,970 | 15,840 |
| 1 „ 2 | 9,680 | 9,560 | 1 „ 2 | 5,740 | 5,190 |
| 2 „ 3 | 5,310 | 5,420 | 2 „ 3 | 3,350 | 3,620 |
| 3 „ 4 | 3,430 | 3,620 | 3 „ 4 | 2,310 | 2,700 |
| 4 „ 5 | 2,280 | 2,370 | 4 „ 5 | 1,980 | 2,060 |
| 5 „ 10 | 4,730 | 4,690 | 5 „ 10 | 5,740 | 5,780 |
| 10 „ 15 | 1,700 | 1,590 | 10 „ 15 | 2,930 | 3,240 |
| 15 „ 25 | 5,360 | 5,630 | 15 „ 25 | 6,950 | 7,960 |
| 25 „ 35 | 7,190 | 7,650 | 25 „ 35 | 6,700 | 7,470 |
| 35 „ 45 | 8,660 | 7,830 | 35 „ 45 | 6,850 | 6,790 |
| 45 „ 55 | 8,540 | 7,360 | 45 „ 55 | 6,570 | 6,400 |
| 55 „ 65 | 8,360 | 8,150 | 55 „ 65 | 8,230 | 8,230 |
| 65 „ 75 | 7,710 | 9,110 | 65 „ 75 | 11,030 | 10,230 |
| 75 „ 85 | 4,420 | 6,460 | 75 „ 85 | 9,880 | 10,310 |
| 85 „ 95 | 940 | 1,870 | 85 „ 95 | 2,650 | 3,700 |
| 95 and upwards | 70 | 160 | 95 and upwards | 130 | 380 |

### No. II.—In 100,000 Living at all Ages.

| | London. | | | Surrey. | |
|---|---|---|---|---|---|
| *Ages.* | *Males.* | *Females.* | *Ages.* | *Males.* | *Females.* |
| 0 to 1 | 587 | 423 | 0 to 1 | 353 | 278 |
| 1 „ 2 | 264 | 221 | 1 „ 2 | 106 | 91 |
| 2 „ 3 | 145 | 122 | 2 „ 3 | 62 | 62 |
| 3 „ 4 | 92 | 83 | 3 „ 4 | 42 | 47 |
| 4 „ 5 | 62 | 54 | 4 „ 5 | 36 | 36 |
| 5 „ 10 | 129 | 108 | 5 „ 10 | 106 | 101 |
| 10 „ 15 | 46 | 42 | 10 „ 15 | 51 | 56 |
| 15 „ 25 | 146 | 130 | 15 „ 25 | 129 | 139 |
| 25 „ 35 | 196 | 176 | 25 „ 35 | 124 | 131 |
| 35 „ 45 | 236 | 181 | 35 „ 45 | 127 | 119 |
| 45 „ 55 | 233 | 170 | 45 „ 55 | 122 | 112 |
| 55 „ 65 | 228 | 175 | 55 „ 65 | 152 | 144 |
| 65 „ 75 | 218 | 210 | 65 „ 75 | 204 | 179 |
| 75 „ 85 | 120 | 149 | 75 „ 85 | 183 | 181 |
| 85 „ 95 | 26 | 43 | 85 „ 95 | 49 | 65 |
| 95 and upwards | 2 | 3 | 95 and upwards | 2 | 6 |

### No. III.—In 100,000 Living at each of following Ages.

| | London. | | | Surrey. | |
|---|---|---|---|---|---|
| *Ages.* | *Males.* | *Females.* | *Ages.* | *Males.* | *Females.* |
| 0 to 1 | 23,420 | 17,905 | 0 to 1 | 14,729 | 10,791 |
| 1 „ 2 | 10,694 | 9,865 | 1 „ 2 | 4,386 | 3,657 |
| 2 „ 3 | 5,331 | 5,164 | 2 „ 3 | 2,284 | 2,328 |
| 3 „ 4 | 3,912 | 3,737 | 3 „ 4 | 1,716 | 1,889 |
| 4 „ 5 | 2,717 | 2,685 | 4 „ 5 | 1,437 | 1,515 |
| 5 „ 10 | 1,237 | 1,144 | 5 „ 10 | 875 | 830 |
| 10 „ 15 | 482 | 466 | 10 „ 15 | 443 | 520 |
| 15 „ 25 | 759 | 619 | 15 „ 25 | 690 | 754 |
| 25 „ 35 | 1,070 | 917 | 25 „ 35 | 816 | 843 |
| 35 „ 45 | 1,788 | 1,377 | 35 „ 45 | 1,135 | 1,051 |
| 45 „ 55 | 2,726 | 2,001 | 45 „ 55 | 1,459 | 1,357 |
| 55 „ 65 | 4,812 | 3,805 | 55 „ 65 | 2,829 | 2,683 |
| 65 „ 75 | 9,185 | 7,827 | 65 „ 75 | 6,389 | 5,460 |
| 75 „ 85 | 18,472 | 16,170 | 75 „ 85 | 14,398 | 6,110 |
| 85 „ 95 | 31,995 | 30,326 | 85 „ 95 | 32,238 | 27,862 |
| 95 and upwards | 37,304 | 39,994 | 95 and upwards | —— | 39,029 |

The first two parts of this table have been calculated from the tables of population and mortality in the ninth annual report of the Registrar-General. The third part of the table, where the mortality is shewn with reference to the numbers living at each of the ages specified, is copied from the ninth annual report of the Registrar-General before-mentioned, where the numbers are ready worked out. Examined in this point of view, it is only in the female sex that the mortality of Surrey rises above that of the metropolis. This takes place between the ages of ten and twenty-five, to a considerable extent, as shown by the table and the diagram by which it is illustrated.* If some of the most unhealthy parts of the metropolis are compared with the more distant and rural part of Surrey, the mortality at the period of life immediately succeeding to puberty, is found to be still more in favour of the town and against the country, comparing it even with the numbers living at that particular age. This is the case in both sexes, but in the most marked degree in the female sex. For instance, the annual deaths in St. Giles', amongst males, between the ages of fifteen and twenty-five, in the seven years 1838 to 1844 inclusive, were .589 per cent. or not quite fifty-nine in 10,000, and in Clerkenwell they were ·769 per cent. or not quite seventy-seven in 10,000; whilst in the registration district comprising Guildford, Farnham, and Hambledon, in Surrey, the mortality was ·775 per cent. or over seventy-seven in 10,000. Amongst females, the annual mortality at this period of life was only sixty-eight in 10,000 in St. Giles', and sixty-three in 10,000 in Clerkenwell; whilst in the Guildford district it was at the rate of ninety in 10,000 living at the same period of life.

In some of the large manufacturing towns, as Manchester and Liverpool, the mortality is high at every period of life, when compared with those living at each period, but in the first ten years after puberty it is low as compared with the total mortality; for instance, the annual deaths in Manchester, in both sexes, between the ages of fifteen and twenty-five, during the seven years alluded to, were 586 in 10,000 deaths at all ages, and in Liverpool 548, whilst in Wiltshire they were 825.

In Manchester and Liverpool, the greatest mortality in any decennial period, subsequent to five years of age is from thirty-five to forty-five in males, the same as in London—that

* Illustrative diagrams were illustrated at the meeting of the Epidemiological Society, at which this paper was read.

is comparing, the deaths at this decennial period with the whole deaths, or with the whole population ; but in females the greatest mortality is from twenty-five to thirty-five, whilst in London it is from sixty-five to seventy-five,—forty years later. In Sheffield it is the same as in Manchester and Liverpool. In Huddersfield the greatest mortality amongst females is also from twenty-five to thirty-five, whilst amongst males it is from sixty-five to seventy-five, the same as amongst males in Surrey. In Leeds the greatest mortality in adults of both sexes is from fifteen to twenty-five, although there are two districts within twenty miles of it, viz., the Pateley Bridge and the Skipton districts, in which the chief mortality is from seventy-five to eighty-five, or sixty years later than at Leeds. I once lived at Pateley Bridge, near to the church, and remember that a great proportion of the funerals were those of old people, who died, as it was said, of old age, without having had any medical attendance. In the ninth annual report there are six Lancashire towns standing in one dreadful row, at page 232, in all of which the greatest mortality in adult life, in both sexes, is between the ages of fifteen and twenty-five, as at Leeds: they are Blackburn, Preston, Chorley, Rochdale, Bury, and Bolton. Stockport and Macclesfield, in Cheshire, are also in the same predicament. At Nottingham, although a manufacturing town, the highest mortality amongst adults is from sixty-five to seventy-five, in both sexes, or fifty years later than in the towns just enumerated. The highest mortality amongst adults in Birmingham is from thirty-five to forty-five in males, and from sixty-five to seventy-five in females, the same as in London, to which it nearly approaches in point of general salubrity.

The circumstance previously alluded to of the lower rate of mortality amongst young persons, in some of the worst districts of London, than in the most salubrious parts of Surrey, is of considerable interest. There has long been a popular impression that living in town is of itself injurious to people of all ages; and there are hundreds of young men with limited means and not much leisure, who spend a good deal, both of money and of time, in getting a little way out of town every evening by omnibus or other means, and returning in the morning, for the benefit, as they suppose, of their health, whilst they are just of the age when the mortality is less in the town than out of it. If they obtained exercise or fresh air by the journey, it might be an advantage; but it is doubtful whether they do.

It is pretty generally believed that the excess of mortality in large towns, over that of rural districts, is chiefly if not altogether due to certain physical conditions, which might be removed by what are called sanitary measures. In fact, this excess of mortality is sometimes spoken of as the removable part of the mortality of the towns. Now, I do not deny either the necessity or utility of well devised measures of sanitary reform; and, in a former paper, I endeavoured to show how such measures might assist in preventing the spread of cholera; but I would suggest that the chief causes of the difference of mortality between the town and the country are not the external physical conditions alluded to. If the excess of deaths in London over those in Surrey depended altogether on chiefly in overcrowding, want of drainage, noxious effluvia, or deficient or bad supply of water, they ought to affect persons of every age, more or less; and although people, might be better able to resist such influences at one period of life, than at another, they cannot be the cause why young men and women are absolutely subject to a lower mortality in Clerkenwell and St. Giles' than in the healthiest parts of the country.

That the higher mortality of towns does not arise merely from living in the towns, is apparent on examining one town with another. From some of the figures given above, it will have been noticed that a very great difference exists in the mortality of various towns. London, which is prodigiously larger than any of the great provincial towns, has a much lower mortality than they have. Bath, Brighton, Wolverhampton, and Norwich, are almost the only towns with 50,000 inhabitants that have a lower mortality than London; and 50,000 is only about two per cent. of the population of this metropolis. In London, the mortality amongst females, between fifteen and twenty-five years of age, is lower than in the rural districts; but in the towns were textile fabrics are manufactured, the mortality at this period is higher. It is most likely that the mortality at every period of life is influenced more by the habits, occupations, and pecuniary circumstances of the people, than by any other causes. Indeed, when the vital statistics of different trades and occupations have been collected, this has been shown to be the case.

By examining the registration returns of the causes of death, we may expect to arrive at the circumstances which influence the mortality in town and country.

The tenth annual report of the Registrar-General contains tables of the causes of death, in the year 1847, for the metropolis and large divisions of the country, arranged according to the ages in the mortality tables in the ninth report, from which I have quoted above. I have compared the table for London with that for the South Midland division, adjoining to London, which comprises part of Middlesex, and the counties of Hertford, Buckingham, Oxford, Northampton, Huntingdon, Bedford, and Cambridge. The population is chiefly rural, but includes that of the towns of Oxford, Cambridge, and Northampton, besides the considerable suburban districts of Brentford and Edmonton.

The diseases which are most fatal in infancy and early childhood, are bronchitis and pneumonia, convulsions, diarrhœa, hydrocephalus, hooping-cough, and the irruptive fevers. On reducing the number of deaths in 1847, from each of these diseases, in London and the South Midland district, to a per centage of the entire population of each place, I find that every one of them is more fatal in London than in the country district, many of them being considerably more than twice as fatal.

The following table shews the deaths from the above-named diseases, under five years of age, in 100,000 of the population in London and the South Midland district respectively.

| | London. | South Midland. |
|---|---|---|
| Bronchitis and Pneumonia | 194 | 108 |
| Convulsions | 99 | 82 |
| Diarrhœa | 66 | 25 |
| Hydrocephalus | 67 | 23 |
| Measles | 73 | 26 |
| Scarlet Fever | 43 | 30 |
| Hooping Cough | 68 | 30 |
| Small Pox | 31 | 3 |

The diseases which cause the mortality between 15 and 25 to be higher in the South Midland district than in London, are typhus and phthisis. The deaths in 1847 from typhus in London were 144 to 100,000 of the population, whilst in the South Midland district they were 169. Between the ages of 15 and 25 they were 19 in London and 34 in the South Midland, or nearly twice as many. The deaths from phthisis at all ages were 319 in London, and 298 in the South Midland district; but between the ages of 15 and 25 these proportions are reversed, being 57 in London and 72 in the South Midlands. It is necessary to consider phthisis in the two sexes separately, for there is a wide difference between London and the country in the proportion in which this disease affects males and females. The deaths amongst males from phthisis in London, in 1847, were 172 in 100,000 of the population, and in females 146; in the South Midland district the numbers were 124 males and 173 females; the proportions being just reversed, and the deaths from phthisis in the county district amongst females being just one more than amongst males in London. On ascertaining the deaths from phthisis in each sex, in town and country, between the ages of 15 and 25, it is evident how much this disease contributes to reverse the ordinary relation of mortality at this period. In London, the numbers are—males, 30; females, 27. In the South Midland district—males, 25; females, 46. So that, in the country district, a considerable proportion of the females who die of phthisis die earlier than the males, and earlier than both males and females in London. It will be recollected that it is more particularly in females that the country mortality rises above that of London at the period just succeeding to puberty. It is chiefly to phthisis that the great mortality previously alluded to, in early adult life, in many of the manufacturing towns, is due.

The great mortality amongst infants in large towns is no doubt very much due to improper nourishment and want of proper attention in general. More children are deprived of breast milk in London than in the country: the vice of dram-drinking, so common amongst women belonging to the working classes in large towns, is almost unknown in rural districts; and this habit must influence such children as are suckled. In the country, also, the people have some traditional knowledge of the way in which an infant ought to be fed, whilst the poor people in large towns give it "anything that is going," to use their own expression, and feed it with the most stimulating and indigestible food. All these causes cannot fail to increase the frequency of convulsions, hydrocephalus, and diarrhœa. Another cause of the great mortality of infants in large towns is that the various infectious diseases are constantly present, and the children nearly always have them whilst young. Hooping cough and measles are much more fatal in infancy than in children a little more advanced; and in rural districts, where these diseases pay only occasional visits, a certain number of the children escape being attacked by them till a period when they are but little dangerous. In proof of the earlier average attack of these infectious diseases in large towns, I may adduce the fact, that the deaths from scarlet fever between the ages of 10 and 20 are much greater in the South Midland district than in London, the proportions being—South Midland 62, and London 38, in 1,000,000 of the population; whilst, under the age of 5, this disease is much more fatal in London than in the South Midlands.

I believe that the reason of the continued fevers which are grouped together under the name of typhus being more fatal in the country than in London, is similar to that just mentioned, only operating in the opposite direction. Typhoid fever is much less fatal in childhood, when it passes under the name of infantile remittent, than in adult life; and one sees it often enough in children to render it probable that a considerable number of the population of towns gain an immunity from it in after life, by having passed through it at an earlier and safer period.

The greater prevalence of phthisis amongst males in London than in the rural districts, may be easily explained by the in-door employments generally followed, as contrasted with the exercise in the open air of the country; but how can we explain the greater mortality of this same disease in the country amongst females? One prevalent theory of the cause of phthisis is, that it is occasioned or promoted by deficient ventilation; and, as far as males are concerned, in town and country, the facts support the theory. But, amongst females, the disease is more fatal in the rural districts than in London; although it cannot be supposed that the females in the country suffer more from confinement in-doors, or want of fresh air, than in London. The causes of phthisis are involved in great obscurity; but my own impression of the probable cause of its great fatality amongst young women in the rural districts is, that it arises from insufficient nourishment. Poverty and misery have at all times been the lot of a great portion of mankind, and they fall most heavily upon those who cannot obtain employment. When a piece of bacon or cheese reaches the cot of the peasant, it must be reserved for the man, to enable him to work for his family, who must often be content with a scanty allowance of meal and potatoes. The boys often get a little work and food at the neighbouring farm, whilst the girls must still remain at home till old enough for domestic service; and the health often gives way at the time when the girl should change into the woman.

I have been able to give but a very brief outline of a very extensive and important subject, but I hope that the labours of the members of the Epidemiological Society will, sooner or later, make up in a great measure for my deficiencies.

